# Comparison of outcome for holmium laser enucleation prostate and Rezum system in benign prostate hyperplasia: a matched pair analysis

**DOI:** 10.1007/s00345-025-05644-y

**Published:** 2025-04-22

**Authors:** Orkunt Özkaptan, Cüneyd Sevinç, Cengiz Çanakcı, Tahsin Batuhan Aydoğan, Oğuz Türkyılmaz, Mahmut Selman Mert, Alkan Çubuk

**Affiliations:** 1 Department of Urology, Health Sciencies University, Kartal Dr. Lutfi Kirdar City Hospital, D100 Güney Yanyol Cevizli, Kartal, Istanbul, 34890 Turkey; 2 Department of Urology, Beylikduzu Medicana Hospital, İstanbul, Turkey; 3 Department of Urology, Bursa Medicana Hospital, Bursa, Turkey

**Keywords:** Benign prostatic hyperplasia, Rezum, Transurethral resection of prostate, Holmium laser enucleation

## Abstract

**Purpose:**

Our aim was to assess the efficacy and safety of Rezum therapy in patients with symptomatic benign prostatic hyperplasia (BPH) in comparison to Holmium laser enucleation of the prostate (HoLEP).

**Methods:**

The retrospective study included 338 patients (204 HoLEP and 134 Rezum) operated on for BPH between January 2020 and November 2024. A 1:1 matching of treated patients (Rezum) to HoLEP patients based on the closest propensity score value was conducted for 131 successful matching pairs. The groups were compared regarding; Age, Prostate volume, PSA, Catheter-dependence, Qmax, PVR, IIEF, IPSS, PGI-I, operation time (OT) and hospital time (HS).

**Results:**

Concerning perioperative data, there was a statistically significant difference between the Rezum and HoLEP groups regarding OT (8.29 ± 1.21 vs. 68.01 ± 31.01 min; *P* < 0.001) and HS (1 vs. 2.61 ± 1.53 days; *P* < 0.001) with higher values for HoLEP. At the same time, the catheter duration was longer in the Rezum group (7.6 ± 2.19 vs. 3.49 ± 1.16 days; *P* < 0.001). In terms of clinical outcomes, there was a statistically significant improvement in Qmax, IPSS and a decrease in PVR at 12 months for the HoLEP group compared to the Rezum group (16.82 ± 2.27 ml/sn vs. 22.14 ± 7.17 ml/sn, *P* < 0.001; 12.1 ± 2.1 vs. 7.1 ± 1.9, *P* < 0.001; 43.05 ± 32.62 ml vs. 29.73 ± 43.73 ml, *P* = 0.006; respectively). PGI-I scores (*p* = 0.620) and antegrade ejaculation (*p* < 0.001) were in favour of Rezum therapy.

**Conclusion:**

The HoLEP technique demonstrates superior functional outcomes compared to Rezum, particularly in cases with larger prostates. However, Rezum is an ejaculation-sparing technique demonstrating high tolerability, and patient satisfaction even in larger prostates.

## Introduction

Benign prostatic hyperplasia (BPH) is the primary cause of lower urinary tract symptoms in males over 50 years of age [1]. Behavioural and medicinal interventions comprise the majority of the initial treatment of lower urinary tract symptoms (LUTS). Nevertheless, surgery is the most effective treatment option if these methods are ineffective. Holmium laser enucleation of the prostate (HoLEP) is an effective, size-independent therapy for BPH, with the benefits of less blood loss and reduced perioperative morbidity [2, 3].

Long-term follow-up reports indicated that HoLEP was at least equally efficient as TURP [4]. HoLEP is the preferred endoscopic procedure and is considered the gold standard for the surgical management of BPH. A major concern associated with HoLEP is the potential for sexual side effects. Retrograde ejaculation is a common consequence of this surgery, with studies indicating a prevalence of 65–90% [5–7]. Despite the favourable results of HoLEP and other endoscopic treatment techniques, minimally invasive options have evolved to achieve similar functional benefits with lower adverse events while preserving antegrade ejaculation, including Rezum. (NxThera Inc., Maple Grove, Minnesota, USA) [8].

Numerous studies have indicated that Rezum effectively achieves long-term reductions in LUTS and improves urinary flow metrics [9–12]. Rezum technology generates cell necrosis in prostatic tissue by delivering thermal energy via water vapour [13]. The American Urological Association guideline proposes Rezum for patients with prostate volumes less than 80 g and for those seeking to maintain sexual function [8]. Two studies indicated favourable results with Rezum, even in larger prostates (≥ 80 g). Nonetheless, no research comparing Rezum’s efficacy to other treatment modalities has been conducted on patients with larger glands (≥ 80 g). Rezum is provided to all patients with BPH in our patient population who qualify for surgery.

To our knowledge, no study compared the outcomes of Rezum and HoLEP in prostates > 50 g. Our aim was to assess the efficacy and safety of Rezum therapy in patients with symptomatic BPH, including those with larger prostates, in comparison to HoLEP.

## Materials and methods

This retrospectively designed study was conducted in our tertiary care centre between November 2022 and March 2024. The study included 338 patients who were diagnosed with BPH and were eligible for surgery. Inclusion criteria were patients aged 50–80 years with prostate volumes of 50–150 mL and with a maximum urinary flow rate [Q max of < 12 mL/s and International Prostate Symptom Score [IPSS] of > 20) who were unresponsive to treatment with alpha-blockers. Patients with a history of urethral stricture, concomitant bladder stones, previous prostate surgery, suspected or diagnosed neurogenic bladder dysfunction, > 300 mL post-void residual volume or uncorrected coagulopathy were excluded from data analysis.

Baseline and demographic data were collected, including age, preoperative medical morbidities, and ASA score. Routine preoperative workup of our institution includes IPSS, International Index of Erectile Function (IIEF), quality of life assessment (QoL), uroflow, urine analysis and culture if needed, prostate-specific antigen (PSA), pelvic ultrasound to estimate post-void residual urine (PVR ≥ 150 ml) and transrectal ultrasound to estimate prostate size. All patients underwent detailed laboratory work-up including complete blood count and renal profile.

Perioperative parameters included operative time (OT), occurrence of perioperative complications, need for blood transfusion, catheter duration and length of hospitalization time (HT).

All interventions were carried out by two surgeons experienced in Rezum or HoLEP surgery with more than 70 cases operated on before. Rezum was employed under local anaesthesia with sedation as a day case, while HoLEP received spinal anaesthesia. Prophylactic antibiotics were given at the time of induction of anaesthesia. After placement of patients in the lithotomy position, diagnostic urethra-cystoscopy was done.

The Rezum system consists of a radiofrequency generator and a single-use transurethral delivery device, incorporating a standard cystoscope lens. In the lithotomy position, radiofrequency current is applied to an inductive coil heater, generating thermal energy from water vapour. This energy is delivered through a retractable needle via emitter holes in the transurethral device, achieving a penetration depth of approximately 10 mm.

The injection was administered 1 cm distal to the bladder neck at the 3 o’clock and 9 o’clock positions. The needle was inserted for a duration of 9 s, retracted, and subsequently delivered to another treatment site located 1 cm distal to the previous site, continuing until reaching the end of the prostatic tissue just proximal to the verumontanum.

Treatment of the median lobe entails the perpendicular angulation of the needle into the tissue. Upon contact with the prostate tissue, steam releases its stored energy, resulting in immediate necrosis of prostate cells due to the concentrated energy affecting the target cell membranes. The body’s natural healing response gradually absorbs the treated tissue, leading to a reduction in prostate size. The quantity of injections is contingent upon the dimensions of the prostate gland. The post-procedure urethral catheterisation protocol ranged from a minimum of 5 days to a maximum of 8 days, depending on the size of the treated prostatic tissue.

A 26-Fr continuous flow laser resectoscope, a laser-fibre stabilizing bridge, a 120 W holmium laser (Multi Pulse, Jena Surgical, Germany), and a 550-µm end-firing laser fibre were utilized. A 26-Fr nephroscope and a tissue morcellator (Jena Surgical, Germany) were used to remove enucleated tissue. All operations were performed using the En-Bloc technique described by Saitta et al. [14].

The efficacy of the procedures was compared with Qmax, PVR, IPSS, and PGI assessments during the postoperative six-month and twelve-month follow-up visits. Postoperative Patient Global Impression of Improvement (PGI-I) was assessed with the PGI-I Scale.20. Moreover, all subjects completed the International Index of Erectile Function 5 (IIEF-5). Retrograde ejaculation was identified through patient history assessment conducted at baseline and 12 months after the surgical procedure.

Complications such as urine retention, gross haematuria, haemorrhage, transient urinary incontinence, urinary tract infection, and testicular epididymitis were monitored in patients from both groups within three months post-surgery.

### Statistical analysis

Statistical analysis was conducted utilising SPSS 25 (IBM Corp, Armonk, NY, USA). PSM was executed utilising the PSM extension procedure within IBM SPSS. A propensity score was allocated to each patient using the nearest neighbour method, along with age, Prostate volume (PV), Qmax, IPSS, PVR, median lobe, and catheter dependence. Each treated patient (Rezum) was matched 1:1 to one patient from HoLEP based on the closest value of the propensity score.

The Kolmogorov–Smirnov, Kurtosis, and Skewness Tests were used to assess the normality of the data. The clinical characteristics of the two groups were compared with the Mann Whitney U and Student t-test for continuous variables and with the Fisher’s Exact or Pearson Chi-Square test for categorical variables. All statistical tests were two-sided, with *p* < 0.05 considered statistically significant.

This study was conducted in compliance with the International Standard for Good Clinical Practice and the principles of the Declaration of Helsinki. The study protocol was approved by the local ethics committee and written consent was obtained from the patients. (Decision No:2024/010.99/10/46, Decision Date:29.11.2024).

## Results

Before matching, 204 HoLEP patients and 134 Rezum patients were eligible (Table 1). We performed PSM for 131 pairs that were matched successfully. Preoperative baseline characteristics such as age, PV, IPSS, Qmax, catheter dependence and median lobe were not statistically significant between the two groups (Table 2).

**Table 1:** Baseline characteristics before PSM

	Rezum	HoLEP	*P*-Value
Patients, N(%)	136	204	
Age, mean ± SD	65.6 ± 7.3	66.13 ± 7.8	0.557
Prostate volume (g), mean ± SD	76.5 ± 28.9	88 ± 40.2	**0.044**
PSA(ng/ml), mean ± SD	3.17 ± 0.47	3.9 ± 1.14	**0.035**
Catheter-dependent pre-operative, N	9 (6.6)	25 (12.3)	0.13
Qmax, mean ± SD	9.52 ± 1.71	7.51 ± 3.44	**0.01**
PVR, mean ± SD	123.5 ± 36.2	129.04 ± 69	0.39
IIEF, mean ± SD	14.7 ± 3.6	12.1 ± 5.6	**< 0.001**
IPSS	23.9 ± 4.2	25.1 ± 4.5	0.71
Median lobe	37 (27.2)	64 (31.4)	0.48
Patients with PV ≥ 80 g, N(%)	52 (38.2)	84 (42.2)	0.27

PVR: Post-void residual urine IIEF: International Index of Erectile Function IPSS: International Prostate Symptom Score PSA: Prostate-specific antigen PV: Prostate volume

**Table 2:** Baseline characteristics after PSM

	Rezum	HoLEP	*P*-value
Patients, *N*	131	131	
Age, mean ± SD	64.7 ± 7.6	65.4 ± 7.4	0.47
Prostate volume (g), mean ± SD	73 ± 25.7	76.2 ± 25.9	0.32
PSA (ng/ml), mean ± SD	2.8 ± 0.67	3.07 ± 1.28	0.66
Qmax, mean ± SD	7.9 ± 1.2	7.6 ± 2.7	0.24
PVR, mean ± SD	125.3 ± 90	129 ± 52	0.68
IPSS	23.3 ± 4.1	24.1 ± 3.7	0.71
Median lobe, N(%)	36 (27.5)	38 (29)	0.784
Catheter-dependent pre-operative, N(%)	7 (5.34)	10 (7.63)	0.62
IIEF, mean ± SD	14.6 ± 3.7	14.2 ± 3.1	0.48
**Patients with PV > 80 g**	**50**	**50**	
Age, mean ± SD	64.9 ± 5.8	65.4 ± 7.4	0.7
Prostate volume (g), mean ± SD	103.3 ± 17.3	107 ± 14.1	0.23
PSA (ng/ml), mean ± SD	2.91 ± 0.65	3.27 ± 1.36	0.6
Qmax(ml/s), mean ± sd	7.4 ± 2,8	7.3 ± 1,1	0.48
PVR (ml), mean ± SD	145.8 ± 72.2	134.2 ± 29.2	0.36
IPSS, mean ± SD	24.2 ± 5.8	25.2 ± 4.4	0.69
Median lobe, N(%)	21(10.5)	22 (11)	0.84
Catheter-dependent pre-operative, N(%)	3 (1.5)	5 (2.5)	0.46
IIEF, mean ± SD	14.6 ± 2.6	14.5 ± 3.1	0.86

PVR: Post-void residual urine IIEF: International Index of Erectile Function IPSS: International Prostate Symptom Score PSA: Prostate-specific antigen

Postoperative complications necessitating endoscopic re-intervention included urethral strictures in 6 patients (4.6%) and bladder neck contractures in 2 patients (1.5%) within the HoLEP group. In the Rezum group, 1 patient presented with a urethral stricture (0.76%). Cystoscopic clot evacuation was performed in 4 (3.0%) patients with PV > 80 in the Rezum cohort and re-intervention due to clot formation was necessary in 3 (2.3%) patients (Table 3). No blood transfusion was required for either technique. Overall two patients received a transurethral resection of the prostate due to persistent symptoms and elevated PVR, while one patient underwent repeat Rezum therapy.

**Table 3:** Perioperative complications

	Rezum	HoLEP	*P*-value
Patients, *N*(%)	131	131	
Bladder neck contracture	0	2 (1.5)	
Urinary retention	15 (11.4)	6 (4.6)	0.04
Urinary infection	12(9.2)	16 (12.2)	0.54
Retrograde ejaculation	7(5.3)	128 (97.7)	**< 0.001**
Transient incontinence	2 (1.5)	36 (27.5)	**< 0.001**
Reintervention	4 (3.1)	3 (2.3)	1
**Patients PV ≥ 80 g**,** N(%)**	**50**	**50**	
Urinary retention	7 (3.5)	3 (1.5)	0.31
Urinary infection	12 (6)	7 (3.5)	0.31
Transient incontinence	1 (2)	19 (38)	**< 0.001**
Retrograde ejaculation	4 (8)	50 (100)	**< 0.001**
Reintervention	4 (3.1)	2(2.3)	0.67

Urinary tract infections occurred in 16 (12.2%) patients in the HoLEP group and 12 (9.2%) patients in the Rezum group (*P* = 0.54). Overall, 36 (27.5%) patients in the HoLEP group experienced transient incontinence, compared to 2 (1.5%) patients in the Rezum group (*P* < 0.01). Preoperative and postoperative IIEF scores did not differ between the groups (Tables 3 and 4). Retrograde ejaculation was observed in 7 (5.34%) patients for Rezum and 124 (94.6%) for HoLEP (*P* < 0.01) (Table 3).

**Table 4:** Comparison of perioperative Paramters and clinical outcomes 12 months after surgery

	Rezum	HoLEP	*P*-Value
Patients, *N*(%)	131	131	
Qmax (ml/s), mean ± SD	16.8 ± 2.3	22.1 ± 7.2	**< 0.001**
IPSS, mean ± SD	12.1 ± 2.1	7.1 ± 1.9	**< 0.001**
PVR (ml), mean ± SD	43 ± 32.6	29.7 ± 43.7	**0.006**
PGI-I	1.85 ± 1.18	1.77 ± 1.25	0.57
Operation time(min), mean ± SD	8.3 ± 1.2	68 ± 31	**< 0.001**
Hospital stay (day), mean ± SD	1	2.6 ± 1.5	**< 0.001**
Catheterisation time, day, mean ± SD	7.6 ± 2.2	3.5 ± 1.2	**< 0.001**
IIEF, mean ± SD	14.9 ± 4.6	14.2 ± 2	0.1
**Patients PV ≥ 80 g**,** N**	**50**	**50**	
Qmax, mean ± SD	14,9 ± 2,3	21,9 ± 6,7	**< 0.001**
IPSS, mean ± SD	12.8 ± 2.6	7.9 ± 2.1	**< 0.001**
PVR (ml), mean ± SD	53.8 ± 46	35 ± 41.4	0.06
PGI-I, mean ± SD	1.9 ± 1.3	1.8 ± 1.1	0.68
Operation time(min), mean ± SD	8.7 ± 0.9	73.2 ± 26	**< 0.001**
Hospital stay, day, mean ± SD	1	2.8 ± 1.3	**< 0.001**
Catheterisation time, day, mean ± SD	7.7 ± 2.3	3.5 ± 1.2	**< 0.001**
IIEF, mean ± SD	14.8 ± 2.8	14.1 ± 3.2	0.19

PVR: Post-void residual urine IIEF: International Index of Erectile Function IPSS: International Prostate Symptom Score PGI-I: Postoperative Patient Global Impression of Improvemsent

Concerning perioperative data, there was a statistically significant difference between the Rezum and HoLEP groups regarding OT (8.3 ± 1.2 vs. 68 ± 31.01 min; *P* < 0.001) and HT (1 vs. 2.6 ± 1.53 days; *P* < 0.001) with higher values for HoLEP. All Rezum procedures were performed in day surgery. At the same time, the catheter duration was longer in the Rezum group (7.6 ± 2.19 vs. 3.5 ± 1.2 days; *P* < 0.001) (Table 4).

Regarding clinical outcomes, there was a statistically significant improvement in Qmax, IPSS and a decrease in PVR at 12 months in favour of HoLEP (16.8 ± 2.3 ml/sec vs. 22.1 ± 7.2 ml/sec, *P* < 0.001; 12.1 ± 2.1 vs. 7.1 ± 1.9, *P* < 0.001; 43 ± 32.6 ml vs. 29.7 ± 43.7 ml, *P* = 0.006; respectively) (Table 4). Fifteen patients (11.4%) of Rezum and 6 (4.6%) of the HoLEP patients failed to urinate spontaneously after withdrawing the catheter (*P* = 0.04) (Table 3). Following a week of catheterisation, all patients could urinate spontaneously.

In subgroup analysis for patients with PV ≥ 80 g, 50 pairs were matched. Demographic statistics of the subgroup are presented in Table 2. The improvement in Qmax rate was significantly higher in the HoLEP group (14.8 ± 2.3mL/sec vs. 21 ± 6.7mL/sec; *P* < 0.001). The mean PVR was 53.8 ± 46 g for HoLEP and 35 ± 41.5 g for Rezum (*P* = 0.057). The change in IPSS was statistically significantly different in favour of HoLEP (12.8 ± 2.7 vs. 7.9 ± 2.1, *P* < 0.001) (Table 4). Twelve patients in the Rezum group remained using alpha-blockers postoperatively, of whom eight had a prostate volume above 80 g. Nonetheless, no patient in the Holep group continued their medication, which was statistically significant (*P* = 0.004 and *P* = 0.006 for subgroup PV ≥ 80 g). In the HoLEP group, 19 (38%) patients experienced transient incontinence, while only 1 (2%) patients in the Rezum group experienced this condition (*P* < 0.01). Retrograde ejaculation occurred in all 50 (100%) of the HoLEP patients, in contrast to 4 (8%) of the Rezum patients (*P* < 0.001).

## Discussion

This study compared perioperative outcomes and twelve-month follow-up data of patients who underwent Rezum or HoLEP. Both techniques achieved favourable outcomes regarding voiding symptoms and subjective satisfaction. However, improvements in voiding symptoms using IPSS and peak flow rate favoured HoLEP. In subgroup analysis for larger prostates, the outcomes were even more relevant. The main benefit of Rezum is preserving antegrade ejaculation.

Rezum technology is a minimally invasive treatment modality that can be performed under oral sedation in most patients. Rezum technology delivers thermal energy via water vapour, which triggers cell necrosis when applied to prostatic tissue [12, 13]. The water vapour is delivered in 9-second injection treatments delivered transurethrally to a maximum of 15 treatments. As a result, the procedure is short and does not need to be performed under anaesthesia. Previous studies reported a mean OT of 3–15 min [15–19]. Our OT was similar to this previous research. In contrast, HoLEP has a steep learning curve and has to be performed under spinal or general anaesthesia. Consequently, the OT and HT were longer for HoLEP in our study population. As can be predicted, Samir et al. found longer HT and OT for B-TURP than Rezum therapy. Shorter HT and OT in Rezum therapy compared to B-TURP was associated with lower costs for Rezum Therapy [20, 21]. Furthermore, lower HT and OT may reduce the workload for surgeons and healthcare providers.

For the majority of our patients, the preservation of sexual function particularly ejaculation post-surgery is a significant concern. Rezum therapy is known to preserve or even improve sexual function [19, 22]. Likewise, we did not see any deterioration in sexual function as evaluated by the IIEF-5 scale; instead, we saw an improvement in IIEF-5, although this was statistically insignificant. Concerning HoLEP, there was no adverse impact on long-term patient-reported sexual function. The IIEF domains of Rezum and HoLEP exhibited no difference compared to the baseline, and the changes in IIEF were statistically stable. This result was consistent with previous studies. Klett et al. showed no change in baseline IIEF-5 scores at twelve and 36 months [23]. Another advantage of Rezum is the preservation of antegrade ejaculation. The rate of retrograde ejaculation was 5.3% for Rezum. This result was concordant with previous studies [19]. In contrast, retrograde ejaculation is a common complication for HoLEP. In our research, retrograde ejaculation was observed in 97.7%, aligning with the literature that reports an incidence ranging from 65 to 90% [5–7]. It should be noted that retrograde ejaculation was the primary concern for our patients when choosing Rezum therapy. The main proportion of our patient population consisted of men who were eligible for HoLEP but sought to preserve ejaculation.

In this trial, HoLEP showed superior outcomes in IPSS, QoL, Qmax and PVR, compared to the Rezum group. However, the average of each parameter exhibited considerable enhancement relative to baseline at 12 months post-treatment in the Rezum group. This outcome was agreed with the results of Tanneru et al., who performed an indirect comparison between TURP and Rezum therapy using randomised controlled trials and found that TURP had more improvement in urinary domain variables than Rezum [24].

The AUA guidelines recommend Rezum therapy in patients with small PV < 80 g and for those wishing to maintain antegrade ejaculation. While they still offer no clear recommendation for Rezum use for those having large prostates [8, 25]. For our patients, PV was not an election criterion for Rezum therapy. Patients preferred Rezum therapy because of the advantage of preserving erectile function and ejaculation. Due to these benefits, it is essential to assess the suitability of Rezum in larger prostates. Only a few studies have investigated this topic [26–28]. In the study by Eltermann et al. IPSS improved by 59%, QoL score improved by 70%, and Qmax improved by 59% at 12 months while erectile and ejaculatory function remained preserved [26]. Similarly, Bole R. et al. found comparable success rates with Rezum for larger prostates [27]. Our study revealed significant improvement in IPSS and Qmax for patients with PV ≥ 80 g; however, HoLEP had a better outcome in IPSS, QoL, Qmax and PVR than the Rezum group. It is important to note although Rezum indicates inferior functional outcomes relative to HoLEP, subjective satisfaction rates (PGI-I) were comparable between the two methods. This may be attributed to the maintenance of antegrade ejaculation.

Two of four patients with PV ≥ 80 g from the Rezum group received HoLEP ≥ due to persistent symptoms and elevated PVR, while two patients got repeat Rezum therapy. Besides these patients, fifteen (11.4%) patients failed after the first trial voiding and needed temporary catheterisation. Our results were comparable to the study by Babar et al., where 12.1% of patients required catheterisation for a mean of 13.7 days after initial voiding [29]. The longer catheterization duration is one of the disadvantages of Rezum therapy. Consistent with prior studies comparing Rezum with TURP and Bipolar techniques, our results indicated a shorter catheterisation time for HoLEP in comparison to Rezum therapy [19]. The primary disadvantages of HoLEP appear to be transient incontinence and retrograde ejaculation. Prolonged HT and anaesthesia requirements were additional drawbacks for HoLEP.

The limitations of our investigation were the relatively small patient cohort. The retrospective design presents selection bias, potentially leading to an underestimation of negative outcomes. Furthermore, the short-term outcomes of the current cohort might indicate another limitation. Another drawback that might be emphasized is the relatively extended hospitalization time and catheter duration. Prospective randomised comparative studies with extended follow-up and larger cohorts are required to confirm our findings. Another weakness is the reliability of the data concerning ejaculation preservation, as the analysis depended on subjective evaluation.

## Conclusion

The HoLEP technique demonstrates superior functional outcomes compared to Rezum, particularly in cases with larger prostates. Rezum is an ejaculation-sparing technique demonstrating high tolerability, safety, and patient satisfaction in the mid-term, marked by the absence of major complications and a minimal retreatment rate. Rezum therapy reduces the burden on surgeons and healthcare providers. Additional studies are required to confirm these findings and possibly expand the current indications to include a larger population.

## Data Availability

No datasets were generated or analysed during the current study.
